# Distribution and rate of lymph node metastasis following neoadjuvant chemoimmunotherapy in non-small cell lung cancer

**DOI:** 10.1186/s12885-026-16407-2

**Published:** 2026-06-25

**Authors:** Jianing Qiu, Haizhou Yue, Suyu Wang, Kun Li, Aimei Peng, Xinnan Xu

**Affiliations:** 1 Department of Thoracic Surgery, Shanghai Pulmonary Hospital, School of Medicine, Tongji University, 507 Zhengmin Road, Shanghai, 200433 China; 2 Shihezi University, School of Medicine, Shihezi, 832002 China; 3 Department of Respiratory Medicine, Shanghai Tenth People’s Hospital, Tongji University, 301 Middle Yanchang Road, Shanghai, 200433 China

**Keywords:** Neoadjuvant chemoimmunotherapy (NCIT), Non-small cell lung cancer, Lymph node metastasis, Tumor location

## Abstract

**Background:**

Neoadjuvant chemoimmunotherapy (NCIT) has emerged as a standard care for resectable non-small cell lung cancer (NSCLC). However, the pattern of lymph node metastasis (LNM) following this treatment remains poorly characterized. This study aimed to elucidate the distribution and rate of LNM after NCIT.

**Methods:**

We retrospectively analyzed 394 NSCLC patients who received NCIT followed by R0 resection at Shanghai Pulmonary Hospital between 2019 and 2022. Pathological data were evaluated to determine LNM distribution and rate, with subgroup analyses performed based on tumor location and histology. Multivariable logistic regression identified independent predictors of nodal downstaging. Kaplan–Meier analyses evaluated survival outcomes based on the extent of lymphadenectomy. Multivariable Cox proportional hazards regression models were performed to identify independent prognostic factors associated with disease-free survival (DFS) and overall survival (OS).

**Results:**

Among the 394 patients, predominant LNM stations were 13R (9.4%), 7 (9.4%), 13L (5.8%), 2R (5.6%), 4R (5.3%), and 10R (5.1%). N1 metastasis rates were generally higher across all lobes, especially at station 13. Notably, right lower lobe tumors exhibited high metastasis rates to the superior mediastinum. Survival analysis demonstrated a trend toward more favorable DFS among patients receiving systematic lymph node dissection (SLND) compared with those undergoing non-SLND (*P* = 0.182). Similarly, patients with ≥ 6 dissected lymph node stations showed a trend toward more favorable DFS compared with those with < 6 dissected lymph node stations (*P* = 0.100); however, neither association reached statistical significance. Furthermore, multivariable Cox regression identified non-adenocarcinoma histological types (squamous cell carcinoma: HR = 0.58, 95% CI: 0.37–0.92, *P* = 0.020; others: HR = 0.34, 95% CI: 0.17–0.70, *P* = 0.004) as independent predictors of improved DFS. For OS, advanced baseline clinical N stage (cN1 vs. cN0: HR = 8.48, 95% CI: 1.07–66.98, *P* = 0.043) was confirmed as an independent risk factor for worse survival, whereas non-adenocarcinoma/non-squamous histology (HR = 0.13, 95% CI: 0.02–0.95, *P* = 0.044) served as an independent protective factor.

**Conclusions:**

Post-NCIT lymph node involvement patterns in NSCLC patients varied according to primary tumor location. SLND and the retrieval of ≥ 6 dissected lymph node stations were associated with favorable DFS trends, although these findings were not statistically significant and require further validation.

**Supplementary Information:**

The online version contains supplementary material available at https://doi.org/10.1186/s12885-026-16407-2.

## Introduction

Lung cancer remains the leading cause of cancer mortality worldwide and one of the most frequently diagnosed malignancies [1]. Non-small cell lung cancer (NSCLC) constitutes approximately 85% of cases, with 20%–35% presenting as locally advanced disease [2, 3]. Based on pivotal trials like NADIM and CheckMate-816, neoadjuvant chemoimmunotherapy (NCIT) followed by surgery is now established as standard care for resectable NSCLC [4, 5].

Despite NCIT markedly improving pathological response rates, postoperative lymph node metastasis (LNM) remains a significant challenge. Studies indicate that even patients achieving a complete pathological response are at risk of recurrence, with nodal recurrence risk increasing with higher ypN (post-therapy pathologic N) stage [6, 7]. Although NCIT substantially reduces the N stage in NSCLC patients, detailed characterization of the pattern and rate of post-NCIT LNM is currently lacking [8, 9]. Therefore, elucidating the distribution and rate of LNM after NCIT is imperative. This knowledge is crucial for guiding thorough intraoperative lymph node dissection and reducing postoperative recurrence.

Consequently, this study aims to characterize the pattern of postoperative LNM in NCIT-treated NSCLC patients. Our objective is to delineate a detailed mapping of LNM, providing a scientific foundation for more comprehensive and precise lymph node dissection.

## Patients and methods

### Patients

**Fig. 1:**
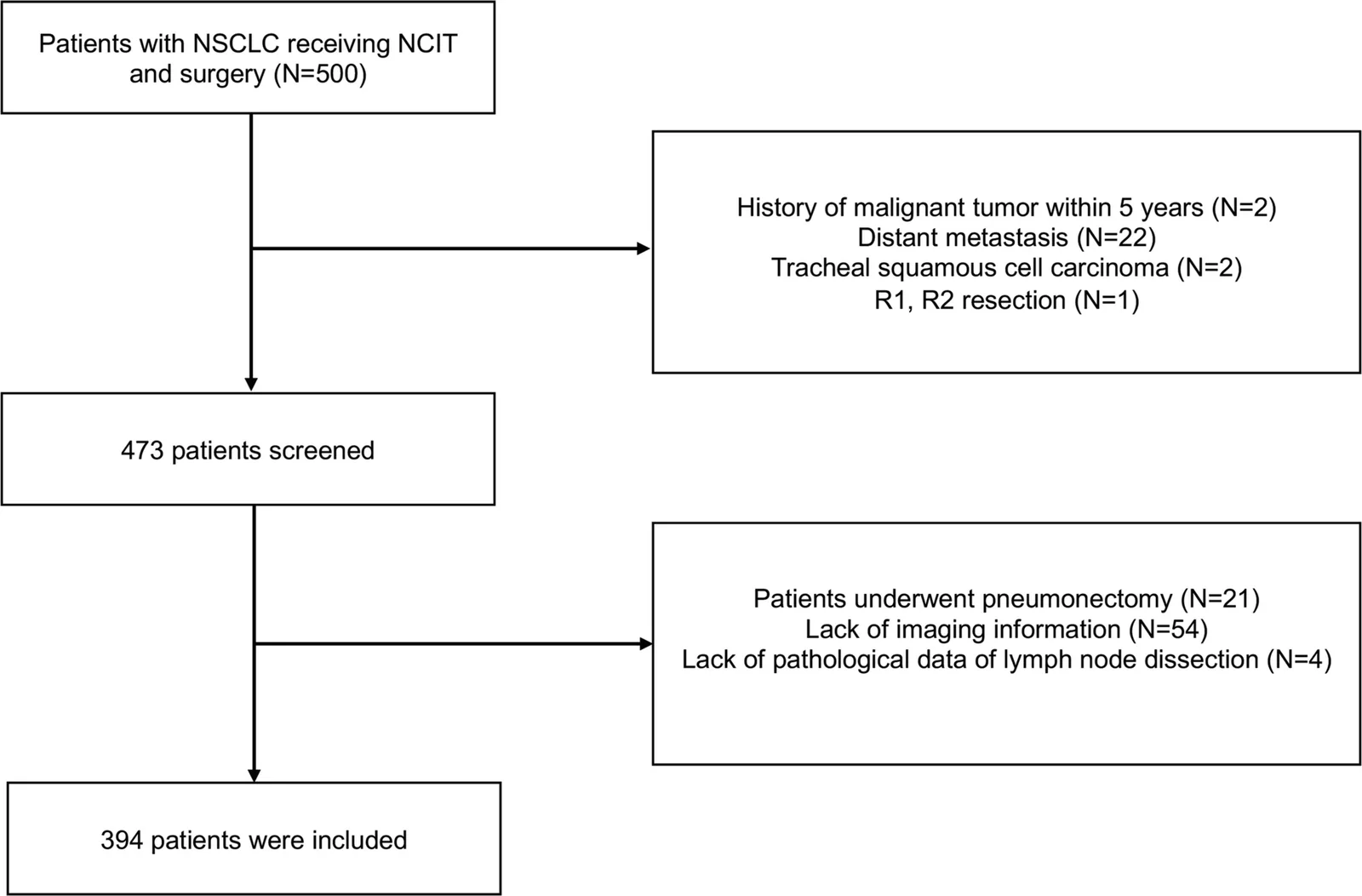
Flow chart

Patients with NSCLC who received NCIT followed by tumor resection at Shanghai Pulmonary Hospital between 2019 and 2022 were retrospectively analyzed. Patients were excluded based on the following criteria: (i) history of other malignancy within 5 years; (ii) R1/R2 resection; (iii) non-NSCLC pathology; (iv) pneumonectomy; (v) distant metastases pre-/intraoperatively; (vi) incomplete clinical data. The final cohort comprised 394 patients (Fig. 1).

All procedures in the present study were performed in accordance with the Declaration of Helsinki as revised in 2024. This retrospective study was approved by the Ethics Review Committee of Shanghai Pulmonary Hospital (No. K25-655). The waiver of informed consent for all participants was specifically reviewed and formally approved by the Ethics Review Committee of Shanghai Pulmonary Hospital due to the retrospective nature of the study.

### Data collection

Patients' medical data, including demographics, preoperative examination, and postoperative pathological information, were retrospectively collected. Tumor staging adhered to the 8th edition TNM classification. Pathological complete response (pCR) was defined as the absence of viable tumor cells in both the primary site and all resected lymph nodes upon histopathological evaluation.

### Endpoints and follow-up

Primary endpoints included Disease-free survival (DFS) and Overall survival (OS). DFS was defined as the duration from surgery to the first instance of tumor recurrence or death. OS was the time interval from the surgery to death from any cause, with surviving patients censored at the last follow-up visit. Survival data were obtained through a combination of electronic medical records and telephone follow-up.

### Lymphadenectomy protocol

The lymphadenectomy protocol strictly adhered to the National Comprehensive Cancer Network (NCCN) Guidelines [10]. Specifically, the adequacy of systematic lymph node dissection (SLND) was defined as the assessment of a minimum of one N1 and three N2 nodal stations. Patients who did not meet this minimum station requirement due to intraoperative clinical constraints were classified as having undergone selective station sampling.

### Classification of the LNM rate

LNM status was assessed through histopathological evaluation of resected specimens. Lymph node stations were documented according to the International Association for the Study of Lung Cancer (IASLC) map, with removal counts recorded per station. The LNM rate was defined as the ratio of the number of lymph node-positive patients at a specific station to the total number of patients in the entire study cohort. A nodal metastasis distribution map was generated using a three-tier color scheme: green (< 5%), orange (5–10%), and red (> 10%). Given the potential influence of variable station sampling and lymph node harvest across different patients in a real-world surgical setting, it should be noted that the term 'LNM rate' used throughout this study operationally refers to the observed postoperative nodal positivity or detection rate, rather than the true absolute metastatic incidence.

### Neoadjuvant chemoimmunotherapy

The neoadjuvant treatment strategy was collaboratively determined by medical oncologists and thoracic surgeons following multidisciplinary team discussion. At our center, treatment typically involves an immune checkpoint inhibitor (ICI), such as nivolumab, pembrolizumab, tislelizumab, sintilimab, camrelizumab, or toripalimab, combined with chemotherapy.

### Statistical analyses

Statistical analyses were performed using R (v4.4.1). Descriptive statistics presented categorical variables as counts (percentages) and continuous variables as median (IQR). Survival curves for DFS and OS were estimated using the Kaplan–Meier method and compared via the log-rank test. The median follow-up duration was determined utilizing the reverse Kaplan–Meier method. Furthermore, univariable and multivariable Cox proportional hazards regression models were employed to identify independent prognostic factors for DFS and OS. Variables demonstrating a trend toward statistical significance (*P* < 0.10) in the univariable analysis were subsequently incorporated into the multivariable models. The optimal cutoff value for the number of dissected lymph node stations was determined based on DFS optimization using the maximally selected rank statistics. Univariable and multivariable logistic regression models were utilized to identify independent predictors of nodal downstaging. All tests were two-sided, with statistical significance set at *P* < 0.05.

## Result

### Baseline characteristics

This study included 394 patients with NSCLC who received NCIT followed by surgery. The cohort comprised 362 males (91.9%) and 32 females (8.1%), with a median age of 64.0 (IQR: 58.0–68.0) years. All patients achieved R0 resection, performed minimally invasively in 294 (74.6%) and via open surgery in 100 (25.4%). SLND was uniformly accomplished in 366 patients (92.9%). Tumor location distribution was: 118 cases right upper lobe (RUL, 29.9%), 7 cases right middle lobe (RML, 1.8%), 46 cases right lower lobe (RLL, 11.7%), 114 cases left upper lobe (LUL, 28.9%), 62 cases left lower lobe (LLL, 15.7%), 43 cases right lower middle lobe (RMLL, 10.9%), and 4 cases right upper middle lobe (RUML, 1.0%). The median number of lymph nodes resected was 17.0 (IQR: 12.0–22.0), and pCR was achieved in 130 patients (33.0%). Regarding nodal staging, 9 of 394 patients (2.3%) were upstaged on postoperative pathological assessment. Specifically, four patients were upstaged from cN0 to pN1, three from cN0 to pN2, and two from cN1 to pN2. Conversely, a large proportion of patients clinically staged as cN1 were downstaged: 47 of 56 cN1 patients (83.9%) were pathologically pN0. Other downstaging events are summarized in Table S1 and Figure S1. Patient characteristics are detailed in Table 1.

**Table 1:** Characteristics of 394 patients

Characteristic	*N = *394
Age	64.0 (IQR: 58.0–68.0)
Sex, n (%)	
Male	362 (91.9%)
Female	32 (8.1%)
Smoking history, n (%)	270 (68.5%)
ECOG, n (%)	
0	165 (41.9%)
1	218 (55.3%)
2	11 (2.8%)
Clinical T status, n (%)	
1	49 (12.4%)
2	112 (28.4%)
3	74 (18.8%)
4	159 (40.4%)
Clinical N status, n (%)	
0	47 (11.9%)
1	56 (14.2%)
2	258 (65.5%)
3	33 (8.4%)
Clinical TNM stage, n (%)	
1	7 (1.8%)
2	28 (7.1%)
3	359 (91.1%)
Surgical type, n (%)	
Open	100 (25.4%)
VATS	261 (66.2%)
RATS	33 (8.4%)
Histology, n (%)	
Adenocarcinoma	112 (28.4%)
Squamous cell carcinoma	238 (60.4%)
Others	44 (11.2%)
Tumor location, n (%)	
Right upper lobe	118 (29.9%)
Right middle lobe	7 (1.8%)
Right lower lobe	46 (11.7%)
Left upper lobe	114 (28.9%)
Left lower lobe	62 (15.7%)
Right middle and lower lobe	43 (10.9%)
Right upper and middle lobe	4 (1.0%)
Pathological T status, n (%)	
0	143 (36.3%)
1	120 (30.5%)
2	100 (25.4%)
3	25 (6.3%)
4	6 (1.5%)
Pathological N status, n (%)	
0	275 (69.8%)
1	41 (10.4%)
2	78 (19.8%)
Pathological stage, n (%)	
pCR	130 (33.0%)
I	111 (28.2%)
II	86 (21.8%)
III	67 (17.0%)
PD-L1 expression, n (%)	
< 1%	80 (20.3%)
1–49%	61 (15.5%)
≥ 50%	37 (9.4%)
Unknown	216 (54.8%)
Gene mutation, n (%)	
No mutation	197 (50.0%)
KRAS	34 (8.6%)
ALK or EGFR	8 (2.0%)
Others	17 (4.3%)
Unknown	138 (35.0%)
Number of examined lymph nodes	17.0 (IQR: 12.0–22.0)
Number of metastatic lymph nodes	0.0 (IQR: 0.0–1.0)
SLND	366 (92.9%)
Tumor length (mm)	28.0 (IQR: 18.0–38.0)

*ECOG* Eastern Cooperative Oncology Group Performance Status, *RATS* Robot-assisted thoracic surgery, *VATS* Video-assisted thoracoscopic surgery, *SLND* Systematic Lymph Node Dissection

### Overall LNM distribution

**Fig. 2:**
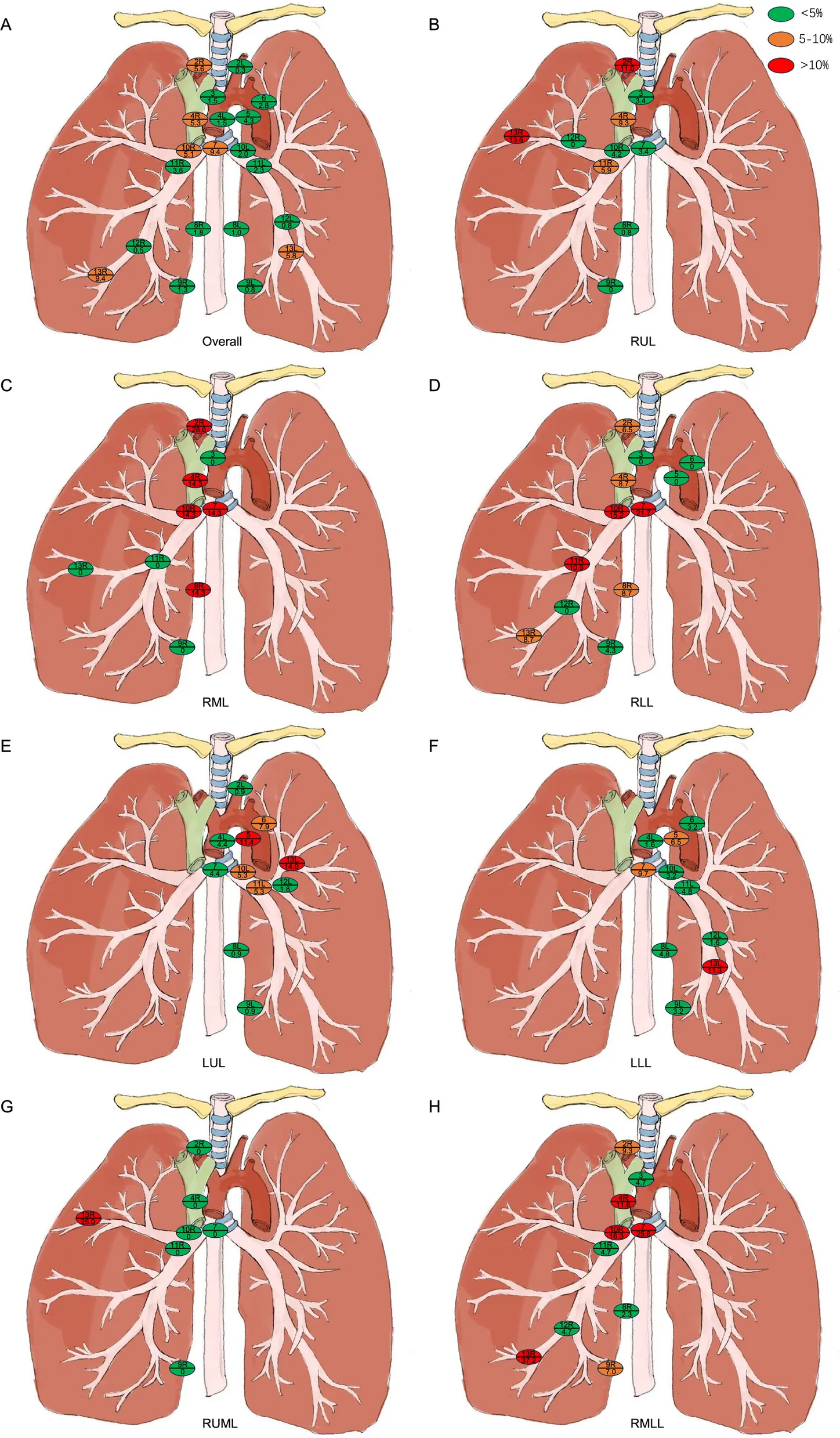
Sites and rate of lymph node metastasis in all patients and in patients with tumors located in different lobes. The LNM is defined as the ratio of the number of lymph node-positive patients at a specific station to the total number of patients in the entire study cohort. **A**. Overall lobes; **B**. RUL: Right upper lobe; **C**. RML: Right middle lobe; **D**. RLL: Right lower lobe; **E**. LUL: Left upper lobe; **F**. LLL: Left lower lobe; **G**. RUML: Right upper middle lobe; **H**. RMLL: Right lower middle lobe

This study analyzed the distribution and rate of LNM in the included patient cohort (Table 2, Fig. 2A). Lymph node stations were categorized anatomically as follows: superior mediastinal (2R, 2L, 3, 4R, 4L), aortic (5, 6), inferior mediastinal (7, 8R, 8L, 9R, 9L), and hilar/intrapulmonary (10R, 10L, 11R, 11L, 12R, 12L, 13R, 13L). Nodes with metastatic rates below 5% (marked green) included 2L (0.3%), 3 (1.5%), 4L (1.5%), 5 (4.3%), 6 (2.8%), 8R (1.8%), 8L (1.0%), 9R (1.3%), 9L (0.8%), 10L (2.0%), 11R (3.6%), 11L (2.3%), 12R (0.5%), and 12L (0.8%). Nodes with metastatic rates between 5 and 10% (marked orange) comprised 2R (5.6%), 4R (5.3%), 7 (9.4%), 10R (5.1%), 13R (9.4%), and 13L (5.8%). Critically, no lymph node station demonstrated a metastatic rate exceeding 10% in this cohort.

**Table 2:** LNM rate overall and according to tumor location in 394 patients

Nodal station	Overall	RUL	RML^*^	RLL	LUL	LLL	RUML^*^	RMLL
2R	5.6 (22/394)	11.0 (13/118)	2/7	6.5 (3/46)	/	/	0/4	9.3 (4/43)
2L	0.3 (1/394)	/	/	/	0.9 (1/114)	/	/	/
3A/3P	1.5 (6/394)	3.4 (4/118)	0/7	0.0 (0/46)	/	/	/	4.7 (2/43)
4R	5.3 (21/394)	9.3 (11/118)	1/7	8.7 (4/46)	/	/	0/4	11.6 (5/43)
4L	1.5 (6/394)	/	/	/	4.4 (5/114)	1.6 (1/62)	/	/
5	4.3 (17/394)	/	/	0.0 (0/46)	11.4 (13/114)	6.5 (4/62)	/	/
6	2.8 (11/394)	/	/	0.0 (0/46)	7.9 (9/114)	3.2 (2/62)	/	/
7	9.4 (37/394)	3.4 (4/118)	1/7	21.7 (10/46)	4.4 (5/114)	9.7 (6/62)	0/4	25.6 (11/43)
8R	1.8 (7/394)	0.8 (1/118)	1/7	8.7 (4/46)	/	/	/	2.3 (1/43)
8L	1.0 (4/394)	/	/	/	0.9 (1/114)	4.8 (3/62)	/	/
9R	1.3 (5/394)	0.0 (0/118)	0/7	4.3 (2/46)	/	/	0/4	7.0 (3/43)
9L	0.8 (3/394)	/	/	/	0.9 (1/114)	3.2 (2/62)	/	/
10R	5.1 (20/394)	4.2 (5/118)	1/7	15.2 (7/46)	/	/	0/4	16.3 (7/43)
10L	2.0 (8/394)	/	/	/	5.3 (6/114)	3.2 (2/62)	/	/
11R	3.6 (14/394)	5.9 (7/118)	0/7	10.9 (5/46)	/	/	0/4	4.7 (2/43)
11L	2.3 (9/394)	/	/	/	5.3 (6/114)	4.8 (3/62)	/	/
12R	0.5 (2/394)	0.0 (0/118)	/	0.0 (0/46)	/	/	/	4.7 (2/43)
12L	0.8 (3/394)	/	/	/	1.8 (2/114)	1.6 (1/62)	/	/
13R	9.4 (37/394)	13.6 (16/118)	0/7	8.7 (4/46)	/	/	1/4	37.2 (16/43)
13L	5.8 (23/394)	/	/	/	14.0 (16/114)	11.3 (7/62)	/	/

The LNM is defined as the ratio of the number of lymph node-positive patients at a specific station to the total number of patients in the entire study cohort

*RUL* Right upper lobe, *RML* Right middle lobe, *RLL* Right lower lobe, *LUL* Left upper lobe, *LLL* Left lower lobe, *RUML* Right upper and middle lobe, *RMLL* Right middle and lower lobe

*Values for RML (*N = *7) and RUML (*N = *4) subgroups are presented as raw counts (number of positive patients/total patients in the subgroup) rather than percentages, to avoid statistical artifact and misinterpretation stemming from extremely limited subgroup sample sizes

### LNM rate in the lung lobes affected

According to the primary tumor site, we analyzed the rate and distribution pattern of LNM (Table 2, Fig. 2). In RUL tumors, station 13R showed the highest metastasis rate (13.6%), followed by station 2R (11.0%) (Fig. 2B). RLL tumors exhibited pronounced involvement at station 7 (21.7%), station 10R (15.2%), and station 11R (10.9%) (Fig. 2D). Among LUL tumors, station 13L demonstrated the highest metastasis rate (14.0%), with station 5 following (11.4%) (Fig. 2E). LLL tumors most frequently involved stations 13L (11.3%) and 7 (9.7%) (Fig. 2F). RMLL tumors showed elevated metastasis rates at station 13R (37.2%), station 7 (25.6%), station 10R (16.3%), and station 4R (11.6%) (Fig. 2H). Due to the extremely limited sample sizes in the RML (*N = *7) and RUML (*N = *4) subgroups, their nodal involvement is presented strictly as raw counts rather than calculated percentages. Consequently, only qualitative descriptions are provided for these groups, warranting validation in larger cohorts. Notably, intrapulmonary lymph nodes (N1) showed frequent involvement regardless of tumor location. Station 13 demonstrated high metastasis rates across most lobes. Upper lobe tumors preferentially metastasized to stations 2, 3, and 4, while lower lobe tumors favored stations 7, 8, and 9. RLL tumors also displayed superior mediastinal involvement. Left-sided tumors tended to have aortic lymph node (stations 5 and 6) metastasis. Lesions spanning multiple lobes showed higher nodal involvement. These findings highlight the importance of tumor localization and lobar drainage patterns in guiding personalized lymphadenectomy strategies.

### LNM rate of different histological types of tumors

**Fig. 3:**
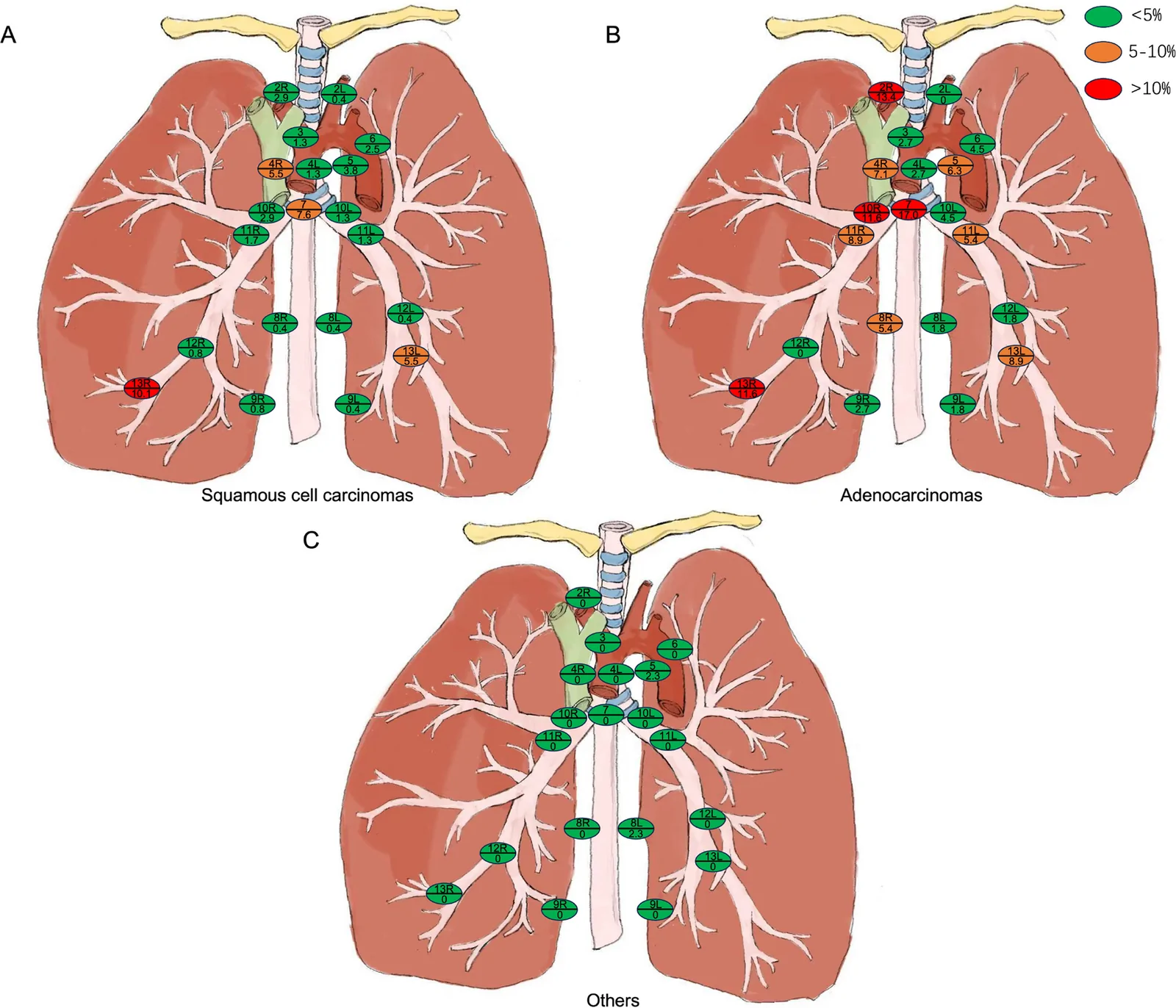
Location and rate of lymph node metastasis in different pathological types of tumors. The LNM is defined as the ratio of the number of lymph node-positive patients at a specific station to the total number of patients in the entire study cohort. **A**. Squamous cell carcinoma; **B**. Adenocarcinoma; **C**. Others

Patients were categorized by histological subtype: squamous cell carcinoma (*N = *238), adenocarcinoma (*N = *112), and other types (*N = *44). Pathological LNM rates across stations between squamous cell carcinoma and adenocarcinoma were systematically compared (Table 3, Fig. 3). Metastases were uncommon in the other types group, observed only at station 5 (2.3%) and station 8L (2.3%) (Fig. 3C). Squamous cell carcinoma exhibited low overall metastasis rates, and station 13R (10.1%) was the only site exceeding 10%, with most stations below 5% (Fig. 3A). In contrast, adenocarcinoma demonstrated significantly higher LNM rates, featuring four stations exceeding 10% and several more between 5 and 10% (Fig. 3B). Compared to squamous cell carcinoma, adenocarcinoma showed numerically greater involvement at station 2R (13.4% vs. 2.9%), station 7 (17.0% vs. 7.6%), station 8R (5.4% vs. 0.4%), station 10R (11.6% vs. 2.9%), station 11R (8.9% vs. 1.7%), and station 11L (5.4% vs. 1.3%). Detailed station-level metastasis rates, raw sample sizes, and exact 95% confidence intervals stratified by histological subtype are summarized in Table S12, with the comprehensive comparison between squamous cell carcinoma and adenocarcinoma visually illustrated in Figure S4. These findings indicate that LNM rate following NCIT is associated with tumor histology.

**Table 3:** Lymph node metastasis rate of different pathological types

Nodal station	Squamous cell carcinomas	Adenocarcinomas	Others
2R	2.9 (7/238)	13.4 (15/112)	0.0 (0/44)
2L	0.4 (1/238)	/	/
3A/3P	1.3 (3/238)	2.7 (3/112)	0.0 (0/44)
4R	5.5 (13/238)	7.1 (8/112)	0.0 (0/44)
4L	1.3 (3/238)	2.7 (3/112)	0.0 (0/44)
5	3.8 (9/238)	6.3 (7/112)	2.3 (1/44)
6	2.5 (6/238)	4.5 (5/112)	0.0 (0/44)
7	7.6 (18/238)	17.0 (19/112)	0.0 (0/44)
8R	0.4 (1/238)	5.4 (6/112)	0.0 (0/44)
8L	0.4 (1/238)	1.8 (2/112)	2.3 (1/44)
9R	0.8 (2/238)	2.7 (3/112)	0.0 (0/44)
9L	0.4 (1/238)	1.8 (2/112)	0.0 (0/44)
10R	2.9 (7/238)	11.6 (13/112)	0.0 (0/44)
10L	1.3 (3/238)	4.5 (5/112)	0.0 (0/44)
11R	1.7 (4/238)	8.9 (10/112)	0.0 (0/44)
11L	1.3 (3/238)	5.4 (6/112)	0.0 (0/44)
12R	0.8 (2/238)	0.0 (0/112)	0.0 (0/44)
12L	0.4 (1/238)	1.8 (2/112)	0.0 (0/44)
13R	10.1 (24/238)	11.6 (13/112)	0.0 (0/44)
13L	5.5 (13/238)	8.9 (10/112)	0.0 (0/44)

The LNM is defined as the ratio of the number of lymph node-positive patients at a specific station to the total number of patients in the entire study cohort

### Sensitivity analysis in the cN+ subpopulation

To verify whether the inclusion of pre-treatment cN0 patients diluted the study findings, a separate analysis was conducted on the cN + subpopulation (Tables S2–S4). Overall, the topographical distribution of LNM remained unchanged, with prominent involvement preserved at stations 13R (10.4%), 7 (10.1%), 4R (5.8%), 13L (5.8%), and 10R (5.5%). After stratification by histological type, the LNM rate of adenocarcinoma remained numerically higher than that of squamous cell carcinoma at stations 2R (15.0% vs. 3.3%), 7 (17.0% vs. 8.6%), 8R (6.0% vs. 0.5%), 10R (12.0% vs. 3.3%), and 11R (10.0% vs. 1.9%). These findings confirm that the inclusion of the 47 cN0 patients did not compromise the validity or clinical guidance of our primary cohort mapping.

### Survival outcomes based on dissected stations

Up to the last follow-up in March 2026, with a median follow-up duration of 36.2 months (95% CI: 34.9–39.5 months) for DFS and 36.0 months (95% CI: 34.8–38.4 months) for OS, we explored the prognostic impact of lymphadenectomy extent and other clinico-pathological variables. Kaplan–Meier analysis showed a numerical difference in DFS between patients undergoing SLND and those without SLND, with a trend toward more favorable DFS in the SLND group; however, this difference did not reach statistical significance (Figure S2A, *P* = 0.182). Similarly, patients with ≥ 6 dissected lymph node stations showed a favorable DFS probability compared with those with fewer dissected stations, although the association was not statistically significant (Figure S3A, *P* = 0.100). As for OS, neither SLND (Figure S2B, *P* = 0.225) nor clearing ≥ 6 stations (Figure S3B, *P* = 0.406) yielded a significant benefit.

### Cox regression analyses of factors associated with survival

To identify independent prognostic factors associated with patient outcomes, univariable and multivariable Cox proportional hazards regression analyses were performed. Variables demonstrating a trend toward statistical significance (*P* < 0.10) in the univariable analysis were subsequently incorporated into the multivariable models. For DFS, the multivariable analysis identified non-adenocarcinoma histology as a robust independent protective factor. Specifically, compared to patients with adenocarcinoma, those with squamous cell carcinoma (HR = 0.58, 95% CI: 0.37–0.92, *P* = 0.020) and other histology (HR = 0.34, 95% CI: 0.17–0.70, *P* = 0.004) exhibited a significantly lower risk of recurrence (Table S5). Regarding OS, the multivariable model revealed that patients presenting with cN1 disease had a significantly higher risk of mortality compared to those with cN0 disease (HR = 8.48, 95% CI: 1.07–66.98, *P* = 0.043). Consistent with the DFS findings, other histology emerged as an independent protective factor for OS, demonstrating a significantly lower risk of mortality compared to adenocarcinoma (HR = 0.13, 95% CI: 0.02–0.95, *P* = 0.044) (Table S6).

### Predictors of lymph node downstaging

Univariable and multivariable logistic regression analyses were performed to identify independent predictors of nodal downstaging in cN + patients (Table S7). Regarding histological subtypes, other histology emerged as a robust independent predictor of successful nodal downstaging (OR = 7.54, 95% CI: 1.64–34.63, *P* = 0.009). In addition, squamous cell carcinoma was more likely to achieve nodal downstaging than adenocarcinoma in the univariable analysis (OR = 1.76, 95% CI: 1.03–3.02, *P* = 0.038), but this association did not reach statistical significance in the multivariable analysis (OR = 1.28, 95% CI: 0.65–2.52, *P* = 0.467). Furthermore, baseline PD-L1 expression and specific driver mutations showed no independent correlation with nodal downstaging.

### Alternative calculation methods

To provide a comprehensive and nuanced anatomical appraisal, we further evaluated the LNM rates using alternative denominator estimation methods. In Supplementary Tables S8 and S9, the LNM rates were calculated as the number of metastatic lymph nodes at each station divided by the total number of lymph nodes resected from that specific station. In addition, we defined the patient-level LNM rate as the ratio of the number of patients with positive lymph nodes at that station to the number of patients who actually underwent resection at that specific station (Supplementary Tables S10 and S11).

## Discussion

This study investigated the LNM distribution and survival outcomes of 394 NSCLC patients following NCIT and subsequent R0 resection. Overall, no lymph node station exceeded a 10% metastasis rate. The most frequently involved LNM sites included stations 2R, 4R, 7, 10R, 13R, and 13L. Histologically, adenocarcinoma demonstrated numerically higher LNM rates than squamous cell carcinoma at stations 2R, 7, 8R, 10R, 11R, and 11L. Furthermore, multivariable analysis identified non-adenocarcinoma/non-squamous histology as independent predictors of both successful nodal downstaging and prolonged OS. Additionally, patients undergoing SLND or those with ≥ 6 dissected lymph node stations showed favorable DFS trends, although these observations did not reach statistical significance.

Existing research in esophageal cancer demonstrates that neoadjuvant therapy reduces LNM frequency and alters its distribution, with different regimens yielding distinct LNM patterns [11–14]. A prior study mapped LNM patterns in 1,308 NSCLC patients who underwent lobectomy without neoadjuvant therapy. Compared to this non-neoadjuvant cohort, our NCIT-treated cohort exhibited a significantly higher metastasis rate in lymph node stations 10–13. Furthermore, within the non-neoadjuvant group, squamous cell carcinoma patients generally showed a higher LNM rate than adenocarcinoma patients. Conversely, this relationship was reversed in our cohort [15]. Gao et al. examined mediastinal nodal involvement in 4,511 NSCLC patients who underwent primary tumor resection without neoadjuvant therapy, revealing LNM distributions and frequencies that differed markedly from our findings [16]. The contrast with our cohort confirms that NCIT may reshape the rate and distribution pattern of LNM.

Previous studies confirm that LNM patterns correlate with primary tumor location. Lower lobe tumors typically show mediastinal LNM confined to the lower mediastinum, rarely involving the upper mediastinum. RUL tumors primarily metastasize to the right upper mediastinum, with infrequent subcarinal involvement. LUL tumors most commonly involve the aortopulmonary window or left upper mediastinum [17–22]. These patterns align substantially with our findings. Nevertheless, our cohort demonstrated a markedly higher rate of superior mediastinal LNM in tumors located in the RLL. This may be attributable to the anatomical pathways of the superficial and deep lymphatic vessels within the pulmonary parenchyma, which converge near the hilum and inferior pulmonary vein, ascending towards the superior mediastinal lymph nodes. Tumor cells have the capacity to bypass the N1 lymph nodes and disseminate to the superior mediastinum, thereby leading to a significant increase in the rate of superior mediastinal LNM [23–25]. Consequently, systematic dissection of superior mediastinal lymph nodes should be considered for RLL cancers to ensure accurate staging and mitigate recurrence risk.

In our cohort, with the exception of the RML (*N = *7), which had a smaller sample size, tumors located in each pulmonary lobe exhibited a high rate of metastasis to station 13 lymph nodes. This phenomenon may be attributed to the pulmonary lymphatic drainage pathway: the intrapulmonary lymphatic vessels are divided into the superficial subpleural plexus and the deep bronchial vascular plexus, both of which initially drain into the parabronchial lymph nodes (13th station) within the same lung segment. Subsequently, lymphatic flow proceeds to the hilum (10th station) and mediastinum via the interlobar lymph nodes (12th and 11th stations). As the 13th station represents the primary level of intrapulmonary lymphatic drainage, tumor cells are more likely to be retained here, leading to a generally elevated metastasis rate [26–28]. Therefore, regardless of tumor location, systematic dissection and thorough pathological evaluation of station 13 lymph nodes are essential during surgery to inform postoperative treatment decisions.

Furthermore, our results demonstrated that adenocarcinoma exhibited numerically higher LNM rates than squamous cell carcinoma at certain nodal stations. In a propensity-matched (PSM) comparison, the mean number of positive lymph nodes (2.2 vs. 0.7; *P* = 0.008) and the rate of LNM (53% vs 29%; *P*= 0.016) were significantly higher in individuals diagnosed with lung adenocarcinoma than in individuals diagnosed with lung squamous cell carcinoma [29]. The underlying biological mechanisms likely involve differential molecular expression. Adenocarcinoma exhibits markedly higher expression of vascular endothelial growth factor C (VEGF-C), which, upon binding VEGFR-3 on lymphatic endothelial cells, activates lymphangiogenesis via the ERK/PI3K-AKT pathway, providing a channel for tumor cells to invade the lymphatic system [30]. Additionally, elevated CXCR4 expression in adenocarcinoma synergizes with VEGF-C to significantly enhance tumor cell metastatic potential [31]. These molecular differences collectively explain the heightened LNM propensity observed in adenocarcinoma. Nevertheless, given the retrospective nature of our study, the baseline staging imbalance remains a notable confounding factor and a study limitation, warranting further risk-adjusted or prospective matched cohorts to fully clarify the independent impact of histology on post-NCIT nodal outcomes.

The prognostic value of the extent of lymphadenectomy following NCIT remains a subject of active debate. In our analysis, patients who underwent SLND exhibited a clinical trend toward improved DFS compared to the non-SLND group (*P* = 0.182; Figure S2A). Similarly, clearing ≥ 6 lymph node stations demonstrated a trend toward better DFS (*P* = 0.100; Figure S3A). Despite these trends, neither strategy yielded a statistically significant OS benefit. This lack of significance is likely attributable to the relatively short follow-up duration within our cohort. These results provide a preliminary signal suggesting that maintaining a relatively comprehensive dissection might potentially mitigate recurrence risks; however, whether broader nodal clearance translates into true survival benefits warrants further validation in larger, multi-center prospective studies with extended follow-up.

In recent years, NCIT has emerged as a key strategy for locally advanced NSCLC. Reported pCR rates in NCIT trials for resectable NSCLC range from 18% to 51.4%: the SAKK 16/14 phase II trial demonstrated 18% pCR; the phase III CheckMate 816 study showed 24% pCR with nivolumab plus platinum-doublet chemotherapy; and a single-center retrospective study of PD-1 inhibitors combined with chemotherapy reported 51.4% pCR [4, 8, 32]. In our cohort, the pCR rate among patients treated with NCIT reached 33%, consistent with published data. These findings confirm that NCIT significantly enhances pathological response rates in NSCLC. Addressing the knowledge gap regarding LNM patterns after NCIT in NSCLC, this study maps priority lymph node stations for dissection. We observed marked variations in metastatic distribution according to tumor location and histological subtype. Characterizing post-NCIT LNM patterns in NSCLC offers critical insights to optimize surgical lymphadenectomy strategies.

To our knowledge, this is the first study to characterize LNM rate and distribution patterns following NCIT for NSCLC. However, several limitations must be acknowledged. Firstly, the single-center design and limited sample size may affect generalizability. Secondly, heterogeneity in therapeutic regimens could influence outcomes. Thirdly, several subgroup analyses, such as those involving RML (*N = *7) and RMUL (*N = *4), were limited by relatively small sample sizes, which may reduce the statistical power and robustness of subgroup-specific inferences. In addition, as this was a retrospective real-world study, variations in lymph node station sampling and the extent of lymph node harvest among individual patients may have introduced potential selection and sampling biases. Therefore, the station-specific nodal findings reported in this study should be interpreted cautiously as observed postoperative nodal positivity/detection rates rather than true metastatic incidence. Future prospective multicenter studies with standardized lymphadenectomy protocols and larger cohorts are warranted to further validate these observed lymph node involvement patterns.

## Conclusion

Post-NCIT lymph node metastasis patterns in NSCLC patients varied according to primary tumor location. Although patients undergoing SLND or with ≥ 6 dissected lymph node stations showed favorable DFS trends, these associations did not reach statistical significance and should be interpreted cautiously. Multivariable analysis identified non-adenocarcinoma/non-squamous histology as an independent factor associated with nodal downstaging and more favorable OS outcome.

## Supplementary Information

Supplementary Material 1.

Supplementary Material 2: Figure S1 Alluvial diagram showing transitions from preoperative clinical N stage (cN) to postoperative pathological N stage (pN). The width of each flow is proportional to the number of patients following that transition. Figure S2 Impact of systematic lymph node dissection on survival outcomes. Kaplan-Meier survival curves for (A) Disease-Free Survival (DFS) and (B) Overall Survival (OS) stratified by the surgical approach (SLND vs. non-SLND) after neoadjuvant chemoimmunotherapy followed by R0 resection. Figure S3 Impact of the extent of lymphadenectomy on survival outcomes. Kaplan-Meier survival curves for (A) Disease-Free Survival (DFS) and (B) Overall Survival (OS) stratified by the number of dissected lymph node stations (≥6 stations vs. <6 stations) after neoadjuvant chemoimmunotherapy followed by R0 resection. Figure S4 Comparison of lymph node metastasis rates across anatomical stations between squamous cell carcinoma and adenocarcinoma. The horizontal barplot displays the station-specific lymph node metastasis rates (%) stratified by histological subtype (squamous cell carcinoma represented in blue; adenocarcinoma represented in red). Error bars represent the exact 95% confidence intervals for each subgroup.

## Data Availability

The data that support the findings of this study are available from the corresponding author upon reasonable request.

## Abbreviations

NCIT: Neoadjuvant chemoimmunotherapy
NSCLC: Non-small-cell lung cancer
LNM: Lymph node metastasis
pCR: Pathological complete response
ypN: Post-therapy pathologic N
IASLC: International Association for the Study of Lung Cancer
ICI: Immune checkpoint inhibitor
RUL: Right upper lobe
RML: Right middle lobe
RLL: Right lower lobe
LUL: Left upper lobe
LLL: Left lower lobe
RMLL: Right lower middle lobe
RUML: Right upper middle lobe
PSM: Propensity-matched
VEGF-C: Vascular endothelial growth factor C
SLND: Systematic Lymph Node Dissection
