# Supplementary material for: Distribution and rate of lymph node metastasis following neoadjuvant chemoimmunotherapy in non-small cell lung cancer
Source: BMC Cancer. 2026 Jun 25;26:1097. doi: 10.1186/s12885-026-16407-2 (PMC13579839; doi:10.1186/s12885-026-16407-2)
Supplement: Supplementary file 1 [file 12885_2026_16407_MOESM1_ESM.docx]

**TableS1** Cross-tabulation of preoperative clinical N stage (cN) and postoperative pathological N stage (pN).

| cN | Overall | pN0 | pN1 | pN2 |
| --- | --- | --- | --- | --- |
| 0 | 47 (100.0%) | 40 (85.1%) | **4 (8.5%)** | **3 (6.4%)** |
| 1 | 56 (100.0%) | 47 (83.9%) | 7 (12.5%) | **2 (3.6%)** |
| 2 | 258(100.0%) | 160 (62%) | 29 (11.2%) | 69 (26.7%) |
| 3 | 33 (100.0%) | 28 (84.8%) | 1 (3%) | 4 (12.1%) |

cN: Clinical N stage; pN:Pathological N status.

**Table S2** Characteristics of 347 cN+ patients

| Characteristic | N = 347 |
| --- | --- |
| Age, yr (median ± SD) | 64.0 (57.0, 68.0) |
| Sex, n (%) |  |
| Male | 318 (91.6) |
| Female | 29 (8.4) |
| Smoking history, n (%) | 240 (69.2) |
| ECOG, n (%) |  |
| 0 | 140 (40.3) |
| 1 | 196 (56.5) |
| 2 | 11 (3.2) |
| Clinical T status, n (%) |  |
| 1 | 49 (14.1) |
| 2 | 101 (29.1) |
| 3 | 61 (17.6) |
| 4 | 136 (39.2) |
| Clinical N status, n (%) |  |
| 1 | 56 (16.1) |
| 2 | 258 (74.4) |
| 3 | 33 (9.5) |
| Clinical TNM stage, n (%) |  |
| II | 11 (3.2) |
| III | 336 (96.8) |
| Surgical type, n (%) |  |
| Open | 84 (24.2) |
| VATS | 231 (66.6) |
| RATS | 32 (9.2) |
| Histology, n (%) |  |
| Adenocarcinoma | 100 (28.8) |
| Squamous cell carcinoma | 209 (60.2) |
| Others | 38 (11.0) |
| Tumour location, n (%) |  |
| Right upper lobe | 105 (30.3) |
| Right middle lobe | 7 (2.0) |
| Right lower lobe | 43 (12.4) |
| Left upper lobe | 96 (27.7) |
| Left lower lobe | 57 (16.4) |
| Right middle and lower lobe | 37 (10.7) |
| Right upper and middle lobe | 2 (0.6) |
| Pathological T status, n (%) |  |
| 0 | 129 (37.2) |
| 1 | 107 (30.8) |
| 2 | 84 (24.2) |
| 3 | 23 (6.6) |
| 4 | 4 (1.2) |
| Pathological N status, n (%) |  |
| 0 | 235 (67.7) |
| 1 | 37 (10.7) |
| 2 | 75 (21.6) |
| Pathological stage, n (%) |  |
| pCR | 117 (33.7) |
| I | 93 (26.8) |
| II | 74 (21.3) |
| III | 63 (18.2) |
| PD-L1 expression, n (%) |  |
| <1% | 75 (21.6) |
| 1-49% | 53 (15.3) |
| ≥50% | 34 (9.8) |
| Unknown | 185 (53.3) |
| Driver mutation status, n (%) |  |
| No mutation | 173 (49.9) |
| KRAS | 31 (8.9) |
| ALK or EGFR | 6 (1.7) |
| Others | 16 (4.6) |
| Unknown | 121 (34.9) |
| SLND | 322 (92.8) |
| Number of examined lymph nodes | 16.0 (IQR: 12.0–22.0) |
| Number of metastatic lymph nodes | 0.0 (IQR: 0.0–1.0) |
| Tumor length (mm) | 28.0 (IQR: 19.0–8.0) |

ECOG: **Eastern Cooperative Oncology Group Performance Status**; RATS: robot-assisted thoracic surgery; VATS: video-assisted thoracoscopic surgery; SLND: Systematic Lymph Node Dissection.

**Table S3** LNM rate overall and according to tumor location in cN+ patients

| Nodal station | Overall | RUL | RML | RLL | LUL | LLL | RUML | RMLL |
| --- | --- | --- | --- | --- | --- | --- | --- | --- |
| 2R | 6.3 | 12.4 | 2/7 | 7.0 | / | / | 0/2 | 10.8 |
| 2L | 0.3 | / | / | / | 1.0 | / | / | / |
| 3A/3P | 1.7 | 3.8 | 0/7 | 0.0 | / | / | / | 5.4 |
| 4R | 5.8 | 10.5 | 1/7 | 9.3 | / | / | 0/2 | 10.8 |
| 4L | 1.7 | / | / | / | 5.2 | 1.8 | / | / |
| 5 | 4.9 | / | / | 0.0 | 13.5 | 7.0 | / | / |
| 6 | 3.2 | / | / | 0.0 | 9.4 | 3.5 | / | / |
| 7 | 10.1 | 3.8 | 1/7 | 20.9 | 5.2 | 8.8 | 0/2 | 29.7 |
| 8R | 2.0 | 1.0 | 1/7 | 9.3 | / | / | / | 2.7 |
| 8L | 1.2 | / | / | / | 1.0 | 5.3 | / | / |
| 9R | 1.4 | 0.0 | 0/7 | 4.7 | / | / | 0/2 | 8.1 |
| 9L | 0.9 | / | / | / | 1.0 | 3.5 | / | / |
| 10R | 5.5 | 4.8 | 1/7 | 14.0 | / | / | 0/2 | 18.9 |
| 10L | 2.3 | / | / | / | 6.3 | 3.5 | / | / |
| 11R | 4.0 | 6.7 | 0/7 | 11.6 | / | / | 0/2 | 5.4 |
| 11L | 2.3 | / | / | / | 5.2 | 5.3 | / | / |
| 12R | 0.6 | 0.0 | / | 0.0 | / | / | / | 5.4 |
| 12L | 0.9 | / | / | / | 2.1 | 1.8 | / | / |
| 13R | 10.4 | 15.2 | 0/7 | 9.3 | / | / | 0/2 | 43.2 |
| 13L | 5.8 | / | / | / | 13.5 | 12.3 | / | / |

RUL: Right upper lobe; RML: Right middle lobe; RLL: Right lower lobe; LUL: Left upper lobe; LLL: Left lower lobe; RUML: Right upper and middle lobe; RMLL: Right middle and lower lobe.

Values for RML (n=7) and RUML (n=2) subgroups are presented as raw counts (number of positive patients / total patients in the subgroup) rather than percentages, to avoid statistical artifact and misinterpretation stemming from extremely limited subgroup sample sizes.

The LNM is defined as the ratio of the number of lymph node-positive patients at a specific station to the total number of patients in the entire study cohort.

**Table S4** Lymph node metastasis rate of different pathological types in cN+patients

| Nodal station | Squamous cell carcinomas | Adenocarcinomas | Others |
| --- | --- | --- | --- |
| 2R | 3.3 | 15.0 | 0.0 |
| 2L | 0.5 | / | / |
| 3A/3P | 1.4 | 3.0 | 0.0 |
| 4R | 5.7 | 8.0 | 0.0 |
| 4L | 1.4 | 3.0 | 0.0 |
| 5 | 4.3 | 7.0 | 2.6 |
| 6 | 2.9 | 5.0 | 0.0 |
| 7 | 8.6 | 17.0 | 0.0 |
| 8R | 0.5 | 6.0 | 0.0 |
| 8L | 0.5 | 2.0 | 2.6 |
| 9R | 1.0 | 3.0 | 0.0 |
| 9L | 0.5 | 2.0 | 0.0 |
| 10R | 3.3 | 12.0 | 0.0 |
| 10L | 1.4 | 5.0 | 0.0 |
| 11R | 1.9 | 10.0 | 0.0 |
| 11L | 1.4 | 5.0 | 0.0 |
| 12R | 1.0 | 0.0 | 0.0 |
| 12L | 0.5 | 2.0 | 0.0 |
| 13R | 11.0 | 13.0 | 0.0 |
| 13L | 5.3 | 9.0 | 0.0 |

The LNM is defined as the ratio of the number of lymph node-positive patients at a specific station to the total number of patients in the entire study cohort

| Characteristics | UnivariateAnalysis | | MultivariateAnalysis | |
| --- | --- | --- | --- | --- |
|  | CI | pValue | CI | pValue |
| Age |  |  |  |  |
| ≥65y vs. <65y | 1.11 (0.78-1.58) | 0.546 |  |  |
| Sex |  |  |  |  |
| Female vs. Male | 1.70 (0.99-2.92) | **0.053** | 1.36 (0.75-2.44) | 0.309 |
| Smoking History |  |  |  |  |
| Yes vs. No | 0.90 (0.62-1.3) | 0.574 |  |  |
| ECOG |  |  |  |  |
| 1 vs. 0 | 0.90 (0.63-1.29) | 0.567 |  |  |
| 2 vs. 0 | 0.46 (0.11-1.88) | 0.277 |  |  |
| cT |  |  |  |  |
| cT2 vs. cT1 | 0.70 (0.41-1.19) | 0.188 | 0.67 (0.39-1.16) | 0.153 |
| cT3 vs. cT1 | 0.60 (0.33-1.11) | 0.103 | 0.65 (0.35-1.21) | 0.172 |
| cT4 vs. cT1 | 0.65 (0.39-1.08) | **0.099** | 0.71 (0.42-1.21) | 0.209 |
| cN |  |  |  |  |
| cN1 vs. cN0 | 1.35 (0.63-2.88) | 0.438 |  |  |
| cN2 vs .cN0 | 1.64 (0.88-3.07) | 0.121 |  |  |
| cN3 vs. cN0 | 1.39 (0.58-3.36) | 0.463 |  |  |
| PD-L1expression |  |  |  |  |
| 1-49% vs. <1% | 1.20 (0.65-2.22) | 0.554 |  |  |
| >=50% vs .<1% | 0.67 (0.28-1.57) | 0.356 |  |  |
| Unknown vs .<1% | 1.40 (0.87-2.27) | 0.168 |  |  |
| Surgical Approach |  |  |  |  |
| VATS vs. Open | 0.90 (0.61-1.34) | 0.605 |  |  |
| RATS vs. Open | 0.82 (0.39-1.71) | 0.600 |  |  |
| Histology |  |  |  |  |
| LUSC vs .LUAD | 0.46 (0.32-0.67) | **<0.001** | 0.58 (0.37-0.92) | **0.020** |
| Others vs. LUAD | 0.32 (0.16-0.66) | **0.002** | 0.34 (0.17-0.7) | **0.004** |
| Driver mutation status |  |  |  |  |
| KRAS vs. No mutation | 1.21 (0.7-2.08) | 0.494 | 0.95 (0.53-1.71) | 0.866 |
| ALK or EGFR vs. No mutation | 1.15 (0.36-3.66) | 0.809 | 0.69 (0.21-2.27) | 0.540 |
| Others vs. No mutation | 1.16 (0.53-2.53) | 0.704 | 0.91 (0.41-2.04) | 0.821 |
| Unknown vs. No mutation | 0.54 (0.35-0.83) | **0.005** | 0.63 (0.4-1.01) | 0.055 |
| SLNMD |  | | | |
| Yes vs. No | 0.68 (0.38-1.21) | 0.185 |  |  |

**Table S5** Cox Proportional Hazards Regression Model for DFS

ECOG: **Eastern Cooperative Oncology Group Performance Status**; RATS: robot-assisted thoracic surgery; VATS: video-assisted thoracoscopic surgery; SLND: Systematic Lymph Node Dissection.

| Characteristics | UnivariateAnalysis | | MultivariateAnalysis | |
| --- | --- | --- | --- | --- |
|  | CI | pValue | CI | pValue |
| Age |  |  |  |  |
| ≥65y vs. <65y | 1.39 (0.79-2.42) | 0.250 |  |  |
| Sex |  |  |  |  |
| Female vs. Male | 1.51 (0.64-3.54) | 0.346 |  |  |
| Smoking History |  |  |  |  |
| Yes vs. No | 1.09 (0.59-1.99) | 0.783 |  |  |
| ECOG |  |  |  |  |
| 1 vs. 0 | 0.66 (0.37-1.16) | 0.148 |  |  |
| 2 vs. 0 | 1.09 (0.26-4.62) | 0.905 |  |  |
| cT |  |  |  |  |
| cT2 vs. cT1 | 1.30 (0.42-4.04) | 0.647 |  |  |
| cT3 vs. cT1 | 2.52 (0.83-7.65) | 0.104 |  |  |
| cT4 vs. cT1 | 1.61 (0.55-4.7) | 0.386 |  |  |
| cN |  |  |  |  |
| cN1 vs. cN0 | 7.87 (1-62.13) | **0.050** | 8.48 (1.07-66.98) | **0.043** |
| cN2 vs .cN0 | 6.68 (0.92-48.78) | **0.061** | 6.55 (0.9-47.84) | 0.064 |
| cN3 vs. cN0 | 8.14 (0.95-69.73) | **0.056** | 8.20 (0.96-70.3) | 0.055 |
| PD-L1expression |  |  |  |  |
| 1-49% vs. <1% | 1.09 (0.39-2.99) | 0.874 |  |  |
| >=50% vs .<1% | 0.76 (0.2-2.87) | 0.687 |  |  |
| Unknown vs .<1% | 1.42 (0.66-3.09) | 0.373 |  |  |
| SurgicalApproach |  |  |  |  |
| VATS vs. Open | 0.79 (0.42-1.46) | 0.443 |  |  |
| RATS vs. Open | 0.83 (0.28-2.51) | 0.748 |  |  |
| Histology |  |  |  |  |
| LUSC vs .LUAD | 0.83 (0.46-1.48) | 0.517 | 0.83 (0.46-1.48) | 0.523 |
| Others vs. LUAD | 0.13 (0.02-0.97) | **0.047** | 0.13 (0.02-0.95) | **0.044** |
| Driver mutation status |  |  |  |  |
| KRAS vs. No mutation | 0.89 (0.31-2.56) | 0.827 |  |  |
| ALK or EGFR vs. No mutation | 1.04 (0.14-7.66) | 0.971 |  |  |
| Others vs. No mutation | 0.84 (0.2-3.57) | 0.819 |  |  |
| Unknown vs. No mutation | 1.04 (0.57-1.9) | 0.909 |  |  |
| SLNMD |  |  |  |  |
| Yes vs. No | 0.59 (0.25-1.39) | 0.230 |  |  |

**Table S6** Cox Proportional Hazards Regression Model for OS

ECOG: **Eastern Cooperative Oncology Group Performance Status**; RATS: robot-assisted thoracic surgery; VATS: video-assisted thoracoscopic surgery; SLND: Systematic Lymph Node Dissection.

**Table S7** Logistic regression analyses of predictors for lymph node downstaging in cN+patients

| Characteristics | Univariate Analysis | | Multivariate Analysis | |
| --- | --- | --- | --- | --- |
|  | CI | *p* Value | CI | *p* Value |
| Age |  |  |  |  |
| ≥65y vs. <65y | 1.20 (0.72-2.00） | 0.480 |  |  |
| Sex |  |  |  |  |
| Female vs. Male | 0.44 (0.20-0.97） | **0.042** | 0.60 (0.21-1.71） | 0.335 |
| Smoking History |  |  |  |  |
| Yes vs. No | 1.56 (0.92-2.64） | 0.099 | 1.43 (0.75-2.72） | 0.277 |
| ECOG |  |  |  |  |
| 1 vs. 0 | 1.49 (0.89-2.50） | 0.128 |  |  |
| 2 vs. 0 | 0.96 (0.24-3.80） | 0.951 |  |  |
| cT |  |  |  |  |
| cT2 vs. cT1 | 1.84 (0.83-4.11） | 0.135 |  |  |
| cT3 vs. cT1 | 1.48 (0.62-3.53） | 0.381 |  |  |
| cT4 vs. cT1 | 1.25 (0.60-2.60） | 0.553 |  |  |
| cN |  |  |  |  |
| cN2 vs. cN1 | 0.52 (0.24-1.13) | 0.098 | 0.64 (0.29-1.41) | 0.267 |
| cN3 vs. cN1 | 8146878.96 (0.00-Inf) | 0.982 | 9333443.46 (0.00-Inf) | 0.980 |
| PD-L1 expression |  |  |  |  |
| 1-49% vs. < 1% | 0.80 (0.36-1.78） | 0.584 |  |  |
| >=50% vs. < 1% | 2.37 (0.73-7.63） | 0.149 |  |  |
| Unknown vs. < 1% | 1.11 (0.59-2.09） | 0.749 |  |  |
| Surgical Approach |  |  |  |  |
| VATS vs. Open | 0.81 (0.43-1.51） | 0.509 |  |  |
| RATS vs. Open | 0.52 (0.21-1.31） | 0.163 |  |  |
| Histology |  |  |  |  |
| LUSC vs. LUAD | 1.76 (1.03-3.02） | **0.038** | 1.28 (0.65-2.52） | 0.467 |
| Others vs. LUAD | 8.47 (1.92-37.38） | **0.005** | 7.54 (1.64-34.63） | **0.009** |
| Driver mutation status |  |  |  |  |
| KRAS vs. No mutation | 2.11 (0.77-5.82） | 0.147 | 2.05 (0.66-6.30） | 0.212 |
| ALK or EGFR vs. No mutation | 0.81 (0.14-4.58） | 0.815 | 1.35 (0.23-7.97） | 0.738 |
| Others vs. No mutation | 0.68 (0.23-1.96） | 0.473 | 1.07 (0.33-3.43） | 0.913 |
| Unknown vs. No mutation | 2.87 (1.53-5.41） | **0.001** | 2.75 (1.39-5.48） | **0.004** |

ECOG: **Eastern Cooperative Oncology Group Performance Status**; RATS: robot-assisted thoracic surgery; VATS: video-assisted thoracoscopic surgery.

**Table S8** LNM rate overall and according to tumor location in 394 patients

| Nodal station | overall (N=394) | | RUL (N=118) | | RML (N=7) | | RLL (N=46) | | LUL (N=114) | | LLL (N=62) | | RUML (N=4) | | RMLL (N=43) | |
| --- | --- | --- | --- | --- | --- | --- | --- | --- | --- | --- | --- | --- | --- | --- | --- | --- |
| 2R | 30/524 | 5.7 | 19/269 | 7.1 | 3/14 | 21.4 | 3/97 | 3.1 | / | / | / | / | 0/12 | 0 | 5/132 | 3.8 |
| 2L | 1/1 | 100.0 | / | / | / | / | / | / | 1/1 | 100 | / | / | / | / | / | / |
| 3A/3P | 6/72 | 8.3 | 4/39 | 10.3 | 0/7 | 0 | 0/14 | 0 | / | / | / | / | / | / | 2/12 | 16.7 |
| 4R | 26/581 | 4.5 | 13/304 | 4.3 | 3/20 | 15.0 | 4/118 | 3.4 | / | / | / | / | 0/13 | 0 | 6/126 | 4.8 |
| 4L | 6/166 | 3.6 | / | / | / | / | / | / | 5/114 | 4.4 | 1/52 | 1.9 | / | / | / | / |
| 5 | 21/276 | 7.6 | / | / | / | / | 0/1 | 0 | 15/181 | 8.3 | 6/94 | 6.4 | / | / | / | / |
| 6 | 12/240 | 5.0 | / | / | / | / | 0/3 | 0 | 9/160 | 5.6 | 3/77 | 3.9 | / | / | / | / |
| 7 | 54/1158 | 4.7 | 5/352 | 1.4 | 1/34 | 2.9 | 15/140 | 10.7 | 6/323 | 1.9 | 6/152 | 3.9 | 0/10 | 0 | 21/147 | 14.3 |
| 8R | 7/205 | 3.4 | 1/108 | 0.9 | 1/7 | 14.3 | 4/29 | 13.8 | / | / | / | / | / | / | 1/61 | 1.6 |
| 8L | 6/109 | 5.5 | / | / | / | / | / | / | 1/68 | 1.5 | 5/41 | 12.2 | / | / | / | / |
| 9R | 7/113 | 6.2 | 0/34 | 0 | 0/1 | 0 | 2/28 | 7.1 | / | / | / | / | 0/2 | 0 | 5/48 | 10.4 |
| 9L | 3/133 | 2.3 | / | / | / | / | / | / | 1/74 | 1.4 | 2/59 | 3.4 | / | / | / | / |
| 10R | 28/458 | 6.1 | 7/245 | 2.9 | 1/11 | 9.1 | 11/101 | 10.9 | / | / | / | / | 0/6 | 0 | 9/95 | 9.5 |
| 10L | 11/387 | 2.8 | / | / | / | / | / | / | 8/257 | 3.1 | 3/130 | 2.3 | / | / | / | / |
| 11R | 25/504 | 5.0 | 13/258 | 5.0 | 0/12 | 0 | 9/115 | 7.8 | / | / | / | / | 0/14 | 0 | 3/105 | 2.9 |
| 11L | 20/369 | 5.4 | / | / | / | / | / | / | 10/218 | 4.6 | 10/151 | 6.6 | / | / | / | / |
| 12R | 2/35 | 5.7 | 0/18 | 0 | / | / | 0/4 | 0 | / | / | / | / | / | / | 2/13 | 15.4 |
| 12L | 3/44 | 6.8 | / | / | / | / | / | / | 2/28 | 7.1 | 1/16 | 6.3 | / | / | / | / |
| 13R | 75/915 | 8.2 | 30/446 | 6.7 | 0/14 | 0 | 6/130 | 4.6 | / | / | / | / | 2/25 | 8.0 | 37/300 | 12.3 |
| 13L | 42/690 | 6.1 | / | / | / | / | / | / | 32/463 | 6.9 | 10/227 | 4.4 | / | / | / | / |

RUL: Right upper lobe; RML: Right middle lobe; RLL: Right lower lobe; LUL: Left upper lobe; LLL: Left lower lobe; RUML: Right upper and middle lobe; RMLL: Right middle and lower lobe.

LNM rate is the proportion of the number of metastatic lymph nodes in each lymph node station in the total number of lymph nodes dissection at that lymph node station.

**TableS9** Lymph node metastasis rate of different pathological types

| Nodal station | Squamous cell carcinomas (N=238) | | Adenocarcinomas (N=112) | | Others (N=44) | |
| --- | --- | --- | --- | --- | --- | --- |
| 2R | 8/311 | 2.6 | 22/177 | 12.4 | 0/36 | 0 |
| 2L | 1/1 | 100 | / | / | / | / |
| 3A/3P | 3/33 | 9.1 | 3/32 | 9.4 | 0/7 | 0 |
| 4R | 15/341 | 4.4 | 11/198 | 5.6 | 0/42 | 0 |
| 4L | 3/109 | 2.8 | 3/36 | 8.3 | 0/21 | 0 |
| 5 | 11/161 | 6.8 | 9/68 | 13.2 | 1/47 | 2.1 |
| 6 | 6/145 | 4.1 | 6/54 | 11.1 | 0/41 | 0 |
| 7 | 24/738 | 3.3 | 30/317 | 9.5 | 0/103 | 0 |
| 8R | 1/117 | 0.9 | 6/67 | 9.0 | 0/21 | 0 |
| 8L | 1/50 | 2.0 | 4/40 | 10.0 | 1/19 | 5.3 |
| 9R | 2/130 | 1.5 | 5/22 | 22.7 | 0/19 | 0 |
| 9L | 1/77 | 1.3 | 2/35 | 5.7 | 0/21 | 0 |
| 10R | 7/267 | 2.6 | 21/153 | 13.7 | 0/38 | 0 |
| 10L | 3/239 | 1.3 | 8/80 | 10.0 | 0/68 | 0 |
| 11R | 6/295 | 2.0 | 19/172 | 11.0 | 0/37 | 0 |
| 11L | 5/220 | 2.3 | 15/92 | 16.3 | 0/57 | 0 |
| 12R | 2/22 | 9.1 | 0/9 | 0 | 0/4 | 0 |
| 12L | 1/30 | 3.3 | 2/11 | 18.2 | 0/3 | 0 |
| 13R | 49/575 | 8.5 | 26/259 | 10.0 | 0/81 | 0 |
| 13L | 25/412 | 6.1 | 17/156 | 10.9 | 0/122 | 0 |

LNM rate is the proportion of the number of metastatic lymph nodes in each lymph node station in the total number of lymph nodes dissection at that lymph node station.

**Table S10** LNM rate overall and according to tumor location in 394 patients

| Nodal station | Overall  (N=394) | | RUL  (N=118) | | RML  (N=7) | | RLL  (N=46) | | LUL  (N=114) | | LLL  (N=62) | | RUML  (N=4) | RMLL  (N=43) | | |
| --- | --- | --- | --- | --- | --- | --- | --- | --- | --- | --- | --- | --- | --- | --- | --- | --- |
| 2R | 22/204 | 10.8 | 13/113 | 11.7 | 2/7 | 28.6 | 3/43 | 7.0 | / | / | / | / | 0/4 | 0 | 4/39 | 10.3 |
| 2L | 1/1 | 100.0 | / | / | / | / | / | / | 1/1 | 100 | / | / | / | / | / | / |
| 3A/3P | 6/34 | 17.6 | 4/20 | 20 | 0/2 | 0 | 0/6 | 0 | / | / | / | / | / | / | 2/6 | 33.3 |
| 4R | 21/208 | 10.1 | 11/111 | 9.9 | 1/7 | 14.3 | 4/46 | 8.7 | / | / | / | / | 0/4 | 0 | 5/40 | 12.5 |
| 4L | 6/79 | 7.6 | / | / | / | / | / | / | 5/52 | 9.6 | 1/27 | 3.7 | / | / | / | / |
| 5 | 17/163 | 10.4 | / | / | / | / | 0/1 | 0 | 13/106 | 12.3 | 4/56 | 7.1 | / | / | / | / |
| 6 | 11/143 | 7.7 | / | / | / | / | 0/1 | 0 | 9/91 | 9.9 | 2/51 | 3.9 | / | / | / | / |
| 7 | 37/386 | 9.6 | 4/113 | 3.5 | 1/7 | 14.3 | 10/45 | 22.2 | 5/113 | 4.4 | 6/61 | 9.8 | 0/4 | 0 | 11/43 | 25.6 |
| 8R | 7/79 | 8.9 | 1/41 | 2.4 | 1/4 | 25 | 4/14 | 28.6 | / | / | / | / | / | / | 1/20 | 5 |
| 8L | 4/48 | 8.3 | / | / | / | / | / | / | 1/27 | 3.7 | 3/21 | 14.3 | / | / | / | / |
| 9R | 5/62 | 8.1 | 0/18 | 0 | 0/1 | 0 | 2/19 | 10.5 | / | / | / | / | 0/1 | 0 | 3/23 | 13.0 |
| 9L | 3/78 | 3.8 | / | / | / | / | / | / | 1/43 | 2.3 | 2/35 | 5.7 | / | / | / | / |
| 10R | 20/198 | 10.1 | 5/104 | 4.8 | 1/7 | 14.3 | 7/42 | 16.7 | / | / | / | / | 0/4 | 0 | 7/41 | 17.1 |
| 10L | 8/155 | 5.2 | / | / | / | / | / | / | 6/100 | 6.0 | 2/55 | 3.6 | / | / | / | / |
| 11R | 14/194 | 7.2 | 7/103 | 6.8 | 0/6 | 0 | 5/41 | 12.2 | / | / | / | / | 0/4 | 0 | 2/40 | 5.0 |
| 11L | 9/157 | 5.7 | / | / | / | / | / | / | 6/101 | 5.9 | 3/56 | 5.4 | / | / | / | / |
| 12R | 2/16 | 12.5 | 0/8 | 0 | / | / | 0/4 | 0 | / | / | / | / | / | / | 2/4 | 50 |
| 12L | 3/26 | 11.5 | / | / | / | / | / | / | 2/16 | 12.5 | 1/10 | 10 | / | / | / | / |
| 13R | 37/199 | 18.6 | 16/109 | 14.7 | 0/3 | 0 | 4/41 | 9.8 | / | / | / | / | 1/4 | 25 | 16/42 | 38.1 |
| 13L | 23/158 | 14.6 | / | / | / | / | / | / | 16/103 | 15.5 | 7/55 | 12.7 | / | / | / | / |

RUL: Right upper lobe; RML: Right middle lobe; RLL: Right lower lobe; LUL: Left upper lobe; LLL: Left lower lobe; RUML: Right upper and middle lobe; RMLL: Right middle and lower lobe.

LNM is defined as the ratio of the number of lymph node positive patients at that station to the number of patients who underwent resection of that station.

**Table S11** Lymph node metastasis rate of different pathological types

| Nodal station | Squamous cell carcinomas (N=238) | | Adenocarcinomas (N=112) | | Others (N=44) | |
| --- | --- | --- | --- | --- | --- | --- |
| 2R | 7/120 | 5.8 | 15/68 | 22.1 | 0/16 | 0 |
| 2L | 1/1 | 100 | / | / | / | / |
| 3A/3P | 3/17 | 17.6 | 3/14 | 21.4 | 0/3 | 0 |
| 4R | 13/123 | 10.6 | 8/68 | 11.8 | 0/17 | 0 |
| 4L | 3/48 | 6.3 | 3/20 | 15.0 | 0/11 | 0 |
| 5 | 9/99 | 9.1 | 7/40 | 17.5 | 1/24 | 4.2 |
| 6 | 6/87 | 6.9 | 5/34 | 14.7 | 0/22 | 0 |
| 7 | 18/234 | 7.7 | 19/109 | 17.4 | 0/43 | 0 |
| 8R | 1/46 | 2.2 | 6/27 | 22.2 | 0/6 | 0 |
| 8L | 1/25 | 4.0 | 2/15 | 13.3 | 1/8 | 12.5 |
| 9R | 2/42 | 4.8 | 3/13 | 23.1 | 0/7 | 0 |
| 9L | 1/44 | 2.3 | 2/22 | 9.1 | 0/12 | 0 |
| 10R | 7/119 | 5.9 | 13/61 | 21.3 | 0/18 | 0 |
| 10L | 3/98 | 3.1 | 5/37 | 13.5 | 0/20 | 0 |
| 11R | 4/117 | 3.4 | 10/61 | 16.4 | 0/16 | 0 |
| 11L | 3/94 | 3.2 | 6/40 | 15.0 | 0/23 | 0 |
| 12R | 2/10 | 20.0 | 0/5 | 0 | 0/1 | 0 |
| 12L | 1/18 | 5.6 | 2/6 | 33.3 | 0/2 | 0 |
| 13R | 24/121 | 19.8 | 13/63 | 20.6 | 0/15 | 0 |
| 13L | 13/96 | 13.5 | 10/38 | 26.3 | 0/24 | 0 |

LNM is defined as the ratio of the number of lymph node positive patients at that station to the number of patients who underwent resection of that station.

**Table S12** Comparison of lymph node metastasis rates between squamous cell carcinoma and adenocarcinoma.

| Nodal station | Squamous cell carcinomaRate (n/N) [95% CI] | AdenocarcinomaRate (n/N) [95% CI] |
| --- | --- | --- |
| 2R | 2.9% (7/238) [1.2% - 6.0%] | 13.4% (15/112) [7.7% - 21.1%] |
| 2L | 0.4% (1/238) [0.0% - 2.3%] | / |
| 3A/3P | 1.3% (3/238) [0.3% - 3.6%] | 2.7% (3/112) [0.6% - 7.7%] |
| 4R | 5.5% (13/238) [2.9% - 9.2%] | 7.1% (8/112) [3.1% - 13.6%] |
| 4L | 1.3% (3/238) [0.3% - 3.6%] | 2.7% (3/112) [0.6% - 7.7%] |
| 5 | 3.8% (9/238) [1.8% - 7.1%] | 6.3% (7/112) [2.6% - 12.5%] |
| 6 | 2.5% (6/238) [0.9% - 5.4%] | 4.5% (5/112) [1.5% - 10.1%] |
| 7 | 7.6% (18/238) [4.5% - 11.7%] | 17.0% (19/112) [10.5% - 25.2%] |
| 8R | 0.4% (1/238) [0.0% - 2.3%] | 5.4% (6/112) [2.0% - 11.3%] |
| 8L | 0.4% (1/238) [0.0% - 2.3%] | 1.8% (2/112) [0.2% - 6.3%] |
| 9R | 0.8% (2/238) [0.1% - 3.0%] | 2.7% (3/112) [0.6% - 7.7%] |
| 9L | 0.4% (1/238) [0.0% - 2.3%] | 1.8% (2/112) [0.2% - 6.3%] |
| 10R | 2.9% (7/238) [1.2% - 6.0%] | 11.6% (13/112) [6.3% - 19.0%] |
| 10L | 1.3% (3/238) [0.3% - 3.6%] | 4.5% (5/112) [1.5% - 10.1%] |
| 11R | 1.7% (4/238) [0.5% - 4.2%] | 8.9% (10/112) [4.4% - 15.8%] |
| 11L | 1.3% (3/238) [0.3% - 3.6%] | 5.4% (6/112) [2.0% - 11.3%] |
| 12R | 0.8% (2/238) [0.1% - 3.0%] | 0.0% (0/112) [0.0% - 3.2%] |
| 12L | 0.4% (1/238) [0.0% - 2.3%] | 1.8% (2/112) [0.2% - 6.3%] |
| 13R | 10.1% (24/238) [6.6% - 14.6%] | 11.6% (13/112) [6.3% - 19.0%] |
| 13L | 5.5% (13/238) [2.9% - 9.2%] | 8.9% (10/112) [4.4% - 15.8%] |
