# Supplementary material for: Distribution and rate of lymph node metastasis following neoadjuvant chemoimmunotherapy in non-small cell lung cancer
Source: BMC Cancer. 2026 Jun 25;26:1097. doi: 10.1186/s12885-026-16407-2 (PMC13579839; doi:10.1186/s12885-026-16407-2)
Supplement: Supplementary file 2 [file 12885_2026_16407_MOESM2_ESM.docx]

Figure S1 Alluvial diagram showing transitions from preoperative clinical N stage (cN) to postoperative pathological N stage (pN). The width of each flow is proportional to the number of patients following that transition.

Figure S2 Impact of systematic lymph node dissection on survival outcomes. Kaplan-Meier survival curves for (A) Disease-Free Survival (DFS) and (B) Overall Survival (OS) stratified by the surgical approach (SLND vs. non-SLND) after neoadjuvant chemoimmunotherapy followed by R0 resection.

Figure S3 Impact of the extent of lymphadenectomy on survival outcomes. Kaplan-Meier survival curves for (A) Disease-Free Survival (DFS) and (B) Overall Survival (OS) stratified by the number of dissected lymph node stations (≥6 stations vs. <6 stations) after neoadjuvant chemoimmunotherapy followed by R0 resection.

Figure S4 Comparison of lymph node metastasis rates across anatomical stations between squamous cell carcinoma and adenocarcinoma. The horizontal barplot displays the station-specific lymph node metastasis rates (%) stratified by histological subtype (squamous cell carcinoma represented in blue; adenocarcinoma represented in red). Error bars represent the exact 95% confidence intervals for each subgroup.
